# Clinical field trial of parenteral amoxicillin for the treatment of clinical and subclinical mastitis in smallholder dairy farms in the upper region of Northern Thailand

**DOI:** 10.14202/vetworld.2023.792-798

**Published:** 2023-04-19

**Authors:** Noppason Pangprasit, Anyaphat Srithanasuwan, Montira Intanon, Witaya Suriyasathaporn, Wasana Chaisri

**Affiliations:** 1 Department of Livestock Clinics, Akkhraratchakumari Veterinary College, Walailak University, Nakhon Si Thamarat 80160, Thailand; 2 Department of Food Animal Clinics, Faculty of Veterinary Medicine, Chiang Mai University, Chiang Mai 50100, Thailand; 3 Research Center of Producing and Development of Products and Innovations for Animal Health, Chiang Mai University, Chiang Mai 50100, Thailand

**Keywords:** bacteriological cure, clinical cure, dairy cow, mastitis, parenteral amoxicillin

## Abstract

**Background and Aim:**

Mastitis, primarily caused by intramammary bacterial infection, is the most expensive disease in the global dairy industry due to its negative impact on milk composition and manufacturing properties. This study aimed to evaluate the efficacy of parenteral amoxicillin in the treatment of clinical and subclinical mastitis in smallholder dairy farms in Northern Thailand.

**Materials and Methods:**

A total of 51 cows with clinical and subclinical mastitis from dairy cooperatives in Lamphun and Chiang Mai provinces, Northern Thailand, were enrolled in this study. Conventional bacteriological procedures were applied to identify the causative bacteria in milk samples from these cows before and 7 days after treatment, and antibiotic susceptibility tests were conducted using the disk diffusion method for all bacteria isolated before treatment. All cows with mastitis were administered 15 mg/kg of amoxicillin (LONGAMOX^®^, Syva Laboratories SA, Spain) intramuscularly every other day for 3 days.

**Results:**

Environmental streptococcal bacteria (*Streptococcus uberis* and *Streptococcus* spp.) were commonly isolated from infected quarters and were highly susceptible to amoxicillin (100%). The clinical efficacy of amoxicillin treatment for clinical mastitis cases was 80.43%, and the bacteriological efficacy was 47.82%, with opportunistic staphylococcal bacteria (coagulase-negative staphylococci) and contagious streptococcal bacteria (*Streptococcus agalactiae*) being the most sensitive microorganisms (100%). In subclinical mastitis cases, the bacteriological efficacy of parenteral amoxicillin was 70.45%, with environmental streptococcal bacteria (*S. uberis*) being the most (100%) sensitive microorganisms.

**Conclusion:**

Amoxicillin is highly efficacious and can be used to treat clinical and subclinical mastitis in dairy cows, particularly mastitis caused by environmental *Streptococcus* spp. These findings could be used to guide treatment regimens in veterinary practice in smallholder dairy farms in Thailand.

## Introduction

Mastitis, which is primarily caused by intramammary (IMM) bacterial infection, is the most expensive disease in the global dairy industry due to its negative effect on milk composition and manufacturing properties [1]. Gram-positive bacteria, either contagious (*Streptococcus agalactiae* or *Staphylococcus aureus*) or environmental (*Streptococcus uberis*, *Streptococcus dysgalactiae*, and *Streptococcus* spp.), are the most common pathogens causing both clinical and subclinical mastitis in several countries [2, 3]. Most treatment regimens depend on the use of antibiotics, such as those belonging to the penicillin and cephalosporin groups that are generally used on dairy farms to prevent and control IMM infection in their herds through IMM or parenteral administration [4, 5]. Intramammary antibiotics are typically prescribed for cows with mild, uncomplicated, or moderate clinical cases [6], whereas parenteral antibiotics are prescribed when udder changes are noticeable or systemic signs are clearly present [7, 8]. The treatment of clinical mastitis is generally performed during lactation, whereas in subclinical cases, antibiotics are not recommended for the treatment during lactation due to high treatment costs and poor efficacy [9]. Although treating subclinical mastitis during lactation is not always economical, several studies have demonstrated that it reduces new infection and transmission, increases the rate of bacteriological cure, and lowers somatic cell count (SCC) [10, 11]. Due to regulations imposing increasingly stringent standards on bulk milk SCC by rejecting milk with high SCC at the milk collection center, the demand for treating cows with subclinical mastitis during lactation is increasing in Thailand.

In terms of treatment regimens, parenteral antibiotics are effective in treating both clinical and subclinical mastitis [10, 12]. The advantage of parenteral antibiotics is that they reduce the risk of infection from infusing antibiotics through the teat canal and enhance drug penetration and diffusion through the udder tissue when swelling is present [12]. Furthermore, they are convenient to use, especially when the infection occurs in more than one quarter. A number of recent studies have demonstrated the efficacy of β-lactamase antibiotics such as penethamate hydroiodide and penicillin G for the treatment of clinical and subclinical mastitis through the parenteral route [12, 13]. According to the previous observations, even penicillin G poorly penetrates into the mammary gland due to its weak acidity and low lipophilic characteristics, although therapeutic doses can be attained in milk due to the very low minimum inhibitory concentration (MIC) values against susceptible organisms [9, 14]. Amoxicillin is an antibiotic used for the treatment of bovine mastitis. It belongs to the semisynthetic extended-spectrum penicillin group, and its mode of action is the inhibition of bacterial cell wall synthesis. When amoxicillin is administered intramuscularly, its concentration in the plasma decreases rapidly due to the distribution of milk into the tissue compartment, similar to that of penicillin. Because amoxicillin has the same pharmacokinetic features as those of parenteral penicillin G, a better efficacy for treating mastitis through the intramuscular route is anticipated [14]. Moreover, amoxicillin, alone or in combination with β-lactamase inhibitors, has been widely used to treat clinical mastitis in dairy cows [15, 16]. According to Roberson *et al*. [15], IMM amoxicillin appeared to be an effective treatment for environmental streptococci in cases of mild or moderate clinical mastitis. De and Mukherjee [16] reported a substantial reduction in SCC and total bacterial count in cows that were provided IMM administrations of 300 mg of amoxicillin + sulbactam during the posttreatment period. Similarly, another study reported a successful clinical recovery of bovine mastitis in lactating cows treated with a combination of amoxicillin and clavulanic acid by IMM infusion [17].

Although numerous studies have been conducted to evaluate the efficacy of IMM amoxicillin in bovine mastitis [15–17], there are scarce investigations on the efficacy of parenteral amoxicillin in treating subclinical and clinical mastitis during lactation. Therefore, this study aimed to evaluate the treatment efficacy of parenteral amoxicillin in clinical and subclinical mastitis in smallholder dairy farms in the upper region of Northern Thailand.

## Materials and Methods

### Ethical approval

This study was approved by the Institutional Animal Care and Use Committee (IAUCC: S3/2558), Faculty of Veterinary Medicine, Chiang Mai University, Thailand.

### Study period and location

The study was conducted from January to June 2019 on two dairy cooperatives in Mae-on (Chiang Mai) and Baan-Ti (Lamphun), both of which are located in the upper region of Northern Thailand.

### Sample collection

A total of 1800 dairy cows from a Thai smallholder dairy farm, defined as a farm with <20 cows (https://region1.dld.go.th) in both cooperatives, were used to determine the sample size using the Epitools program (www.epitool.net) with 95% confidence interval and 2.5% precision. A total of 143 quarters of 51 Holstein Friesian cows with naturally occurring clinical and subclinical mastitis were enrolled in this study. Clinical mastitis cases were identified by the ruminant veterinary practitioner in the quarter with visibly abnormal milk in terms of color, viscosity, or consistency, as well as with or without abnormal udder characteristics, including swelling, heat, pain, or redness. Cows with clinical mastitis and having systemic symptoms were not eligible for inclusion. Subclinical mastitis cases were identified using the California mastitis test (CMT). Briefly, milk from each quarter was stripped into the CMT paddle, and then, an equal volume of CMT test reagent was added and gently agitated for 15 s. The reaction is scored on a scale of 0 (the mixture remains unchanged) to 3 (an almost-solid gel forms). Quarters with no visible abnormal milk and an udder with a CMT score of >1 were diagnosed as having subclinical mastitis [18]. Cows with clinical mastitis and subclinical mastitis with CMT scores 2 and 3 were administered 15 mg/kg of amoxicillin trihydrate (LONGAMOX^®^, Syva Laboratories SA, Spain) intramuscularly every other day for three days that were injected by the ruminant veterinary practitioner. For the microbiological analysis, duplicate milk samples were aseptically collected before and after the last treatment. A total of 10 mL of milk samples were collected aseptically from each quarter using standard protocols described by the National Mastitis Council [19]. All samples were placed at 4°C and immediately transferred to the Central Laboratory of Faculty of Veterinary Medicine, Chiang Mai University, within 2 h–4 h.

### Bacterial isolation and identification

Mastitis pathogens from the milk samples were isolated and identified using the conventional method and a biochemical test. Briefly, 10 μL of milk sample was streaked on 5% bovine blood agar plates and incubated at 37°C for 24 h–48 h. Mastitis pathogens were identified by Gram staining, hemolytic pattern, colony morphology, coagulase test, catalase test, and biochemical tests according to the National Mastitis Council guideline. Contaminated samples were identified as those that contained three or more species of bacteria and were excluded from the analysis [19].

### Antimicrobial susceptibility test

The antimicrobial susceptibility test was conducted in duplicate using the disk diffusion method. All bacterial strains were suspended in 9 mL of 0.85% normal saline solution with the equivalent of 0.5 McFarland turbidity standard (concentration 10^7^–10^8^ colony-forming unit/mL) [20] and spread uniformly with a sterile cotton swab over Mueller–Hinton agar or 5% bovine blood agar. The disks were impregnated with standardized concentrations of the following seven antimicrobial classes commonly used in veterinary practice: amoxicillin (10 μg), cephalexin (30 μg), cloxacillin (5 μg), enrofloxacin (5 μg), gentamicin (10 μg), penicillin G (10 μg), and tetracycline (10 μg) (antimicrobial susceptibility disks, Oxoid, Thermo Scientific™, England). Antimicrobial activity was determined by observing the formation of an inhibitory zone surrounding the antibiotic disk. The zone of inhibition was measured in millimeters using Vernier calipers. Sensitivity to exposed agents was interpreted as susceptible or resistant according to the Clinical and Laboratory Standards Institute [21].

### Definition of clinical and bacteriological cure

Therapeutic effectiveness was evaluated on the basis of clinical and bacteriological cure. Clinical cure was determined by the absence of abnormal milk and udder appearances, such as clots or flakes in the milk and redness or swelling of the udder after a period of 7 days. Bacteriological cure was defined as the absence of growth of the previously isolated pathogens after 1 week of the last amoxicillin administration.

## Results

**Figure-1:**
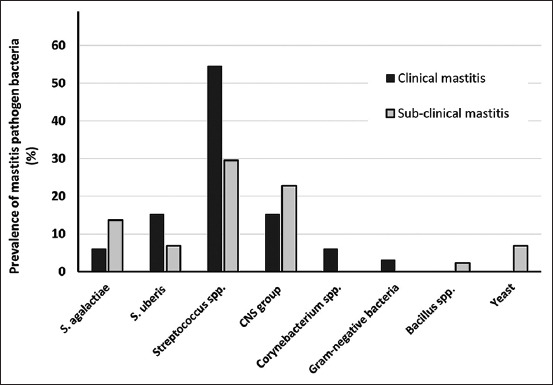
Prevalence of pathogenic bacteria isolated from clinical and subclinical bovine mastitis quarters.

Among the 143 bovine mastitis quarters, there were 45 clinical mastitis quarters (31.46%) and 98 subclinical mastitis quarters (68.53%). Figure-1 shows the different bacterial species isolated from clinical and subclinical mastitis cases before treatment. The majority of clinical mastitis quarters were infected with environmental *Streptococcus* spp. (54.54%), *S. uberis* (15.15%), coagulase-negative staphylococci (CNS) (15.15%), *S. agalactiae* (6.06%), *Corynebacterium* spp. (6.06%), and another group, especially Gram-negative bacteria (3.03%), respectively. Only 13 quarters (13/45, 28.89%) had no microbial growth. Using the CMT, subclinical mastitis was observed in 44 quarters (44.89%), with most quarters being infected with *Streptococcus* spp. (29.54%), followed by CNS (22.73%) and *S. uberis* (6.82%). No bacterial culture growth was observed in 54 quarters (54/98, 55.10%). Other microorganisms such Gram-negative bacteria, yeast, and *Bacillus* spp. were seldom recognized as mastitis pathogens in this area.

The antimicrobial susceptibility characteristics of the bacterial pathogens isolated from clinical and subclinical mastitis quarters are shown in Table-1. Among the seven antimicrobial agents, cloxacillin and tetracycline exhibited low efficacy, causing high bacterial resistance (37.5%–44.45%), whereas amoxicillin, cephalexin, cloxacillin, enrofloxacin, gentamicin, and penicillin exhibited high efficacy, causing high bacterial sensitivity. Among all isolated microorganisms, *S. uberis* exhibited a high level of resistance to tetracycline, with the resistance rate being 50%. All environmental streptococcal bacteria, including *S. uberis* and *Streptococcus* spp., were susceptible to amoxicillin and penicillin, whereas *S. agalactiae* was less susceptible (87.50%).

**Table-1:** The antimicrobial susceptibility profiles of the bacterial pathogens isolated from clinical and subclinical bovine mastitis cases in smallholder dairy farms in Northern Thailand.

Antibiotics	All pathogens (n = 72)	Contagious group		Environmental streptococcal group				Opportunistic bacterial group			
		
		*Streptococcus agalactiae* (n = 8)		*Streptococcus uberis* (n = 8)		*Other environmental Streptococcus* spp. (n = 31)		Coagulase-negative staphylococci (n = 15)		Others (n = 28)	
					
	Susceptible (%)	Susceptible n (%)	^1^MDR	Susceptible n (%)	^1^MDR	Susceptible n (%)	^1^MDR	Susceptible n (%)	^1^MDR	Susceptible n (%)	^1^MDR
Amoxycillin	87.50	7 (87.50)		8 (100.00)		31 (100.00)		10 (66.70)		6 (21.43)	
Cephalexin	93.05	8 (100.00)		8 (100.00)		31 (100.00)		12 (80.00)		8 (28.57)	
Cloxacillin	55.55	7 (87.50)		8 (100.00)		29 (93.55)		10 (66.70)		6 (21.43)	
Enrofloxacin	94.44	8 (100.00)	2 (12.5)	8 (100.00)	0 (0.00)	29 (93.55)	1 (3.20)	13 (86.70)	7 (46.67)	10 (35.71)	4 (14.28)
Gentamicin	86.11	8 (100.00)		8 (100.00)		25 (80.60)		13 (86.70)		8 (28.57)	
Penicillin	84.72	7 (87.50)		8 (100.00)		31 (100.00)		9 (60.00)		6 (21.43)	
Tetracycline	62.50	5 (62.50)		4 (50.00)		22 (71.20)		9 (60.00)		5 (17.85)	

1 MDR: Multidrug-resistant

**Figure-2:**
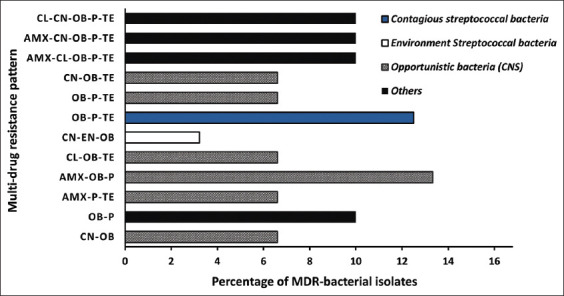
The percentage of bacterial pathogens with multidrug resistance patterns isolated from mastitis cases. AMX: Amoxicillin, CL: Cephalexin, CN: Gentamicin, EN: Enrofloxacin, OB: Cloxacillin, P: Penicillin, TE: Tetracycline

The isolates that were resistant to two or more antibiotics were labeled as multidrug-resistant (MDR) [22]. All MDR isolates were detected in both clinical and subclinical mastitis cases. As shown in Table-1, *S. uberis* did not exhibit MDR, whereas two isolates of *S. agalactiae* (12.50%) and one isolate of the other *Streptococcus* spp. (3.20%) were resistant to more than two types of antibiotics. The MDR pattern is illustrated in Figure-2; *S. agalactiae* as a contagious streptococcus exhibited resistance to tetracycline, cloxacillin, and penicillin. Resistance to cloxacillin was detected in all MDR streptococcal isolates; however, they were sensitive to amoxicillin and cephalexin. In the CNS group, seven MDR staphylococcal isolates (46.67%) were completely resistant to all antibiotics, except enrofloxacin. Furthermore, MDR staphylococcal isolates were the most resistant to cloxacillin, and only two isolates exhibited a susceptible profile to cloxacillin.

Table-2 shows the efficacy of parenteral amoxicillin in both clinical and subclinical mastitis cases. In 46 quarters that were diagnosed with clinical mastitis, 37 quarters (80.43%) were clinically cured. This was determined when the udder and milk sample returned to their normal appearance and there were no abnormalities present. The bacteriological cure was 47.82%. In clinical mastitis quarters, 100% clinical and bacteriological cure was observed in quarters infected with *S. agalactiae* and CNS, with the exception of quarters infected with *S. uberis*, which showed only 80% bacteriological cure. Of 44 quarters diagnosed with subclinical mastitis, 31 quarters were successfully treated and cured (70.45%). *S. uberis* exhibited sensitivity to amoxicillin, as determined by a 100% bacteriological cure rate, followed by *Corynebacterium* spp. (87.50%) and CNS group (80%).

**Table-2:** The efficacy of parenteral amoxicillin in both clinical and subclinical mastitis cases.

Pathogens	Clinical			Subclinical	
	
	n	Clinical cure %	Bacteriological cure %	n	Bacteriological cure %
*Bacillus* spp.	0	0 (0.00)	0 (0.00)	1	0 (0.00)
*Corynebacterium* spp.	2	1 (50.00)	1 (50.00)	8	7 (87.5)
Coagulase-negative staphylococci	5	5 (100.00)	5 (100.00)	10	8 (80)
Gram-negative	1	1 (50.00)	0 (0.00)	0	0 (0.00)
*Streptococcus agalactiae*	2	2 (100.00)	2 (100.00)	6	4 (66.67)
*Streptococcus* spp.	18	14 (77.78)	10 (55.56)	13	8 (61.54)
*Streptococcus uberis*	5	5 (100.00)	4 (80.00)	3	3 (100)
Yeast	0	0 (0.00)	0 (0.00)	3	1 (33.33)
No growth	13	9 (69.23)	0 (0.00)	0	0 (0.00)

## Discussion

The policy for controlling milk quality in Thailand is stringent in order for the country’s dairy industry to reach global standards. Thai farmers primarily concentrate on reducing production costs and enhancing milk quality. Typically, milk collection centers reject raw milk based on criteria with a high SCC. To control and minimize SCC, a variety of antibiotics are commonly used in all cases of mastitis occurring in farms [23].

According to our findings, environmental streptococci, particularly *Streptococcus* spp., were the most common pathogen causing mild-to-moderate clinical and subclinical mastitis, which is consistent with the study of Leelahapongsathon *et al*. [24]. Streptococcal mastitis has also been reported in several countries. In 2015–2019, *Streptococcus* spp. were the most often isolated bacteria from milk samples in northeastern Poland [25], which is similar to the high prevalence of streptococcal infection and high antibiotic resistance observed in Chinese dairy cows with clinical mastitis [26]. Consequently, its significance to udder health has grown in recent decades.

In the present study, resistance to cloxacillin and tetracycline was the highest among the isolated pathogens based on the results of antibacterial susceptibility tests. This could be because cloxacillin is one of the most regularly used IMM infusion drugs for the treatment of mastitis [27]. Moreover, we observed that all mastitis microorganisms, especially *S. uberis* (50%), exhibited very high resistance to tetracycline, which may be due to its long-term parenteral administration for the treatment of anaplasmosis, respiratory disease, and metritis on Thai dairy farms [28]. A similar resistance rate was observed in China, where 59%–98% of *Streptococcus* spp. isolates were resistant to tetracycline [29]. Furthermore, Minst *et al*. [30] and Kabelitz *et al*. [31] reported that *Streptococcus* spp. isolated from bovine mastitis cases exhibited the highest rate of resistance to tetracycline and erythromycin, with 38.50% of streptococcal Group C, 43% of streptococcal Group D, and 46% of streptococcal Group B being resistant to tetracycline. In the present study, both contagious and environment streptococcal bacteria were found to be the most amoxicillin-sensitive pathogens. Various streptococcal species exhibited different categories of sensitivity to amoxicillin, which was consistent with the findings of Bengtsson *et al*. [32], who demonstrated that *S. agalactiae* was more sensitive and responsive to antibiotic treatment, particularly the β-lactamase group. In comparison to Käppeli *et al*. [33], they reported that *S. uberis* exhibited a high degree of sensitivity to penicillin [34], with no clinical resistance observed. All MDR streptococcal isolates were resistant to cloxacillin but susceptible to amoxicillin and cephalexin. Based on the antimicrobial classification, the streptococcal group was sensitive to β-lactamase and showed much greater sensitivity to amoxicillin. Cloxacillin is currently the drug most frequently used in drying and lactation therapy [35]. This is in agreement with Phophi *et al*. [36], who reported differences in the MIC values of the β-lactamase group, including cloxacillin and oxacillin, among streptococcal mastitis pathogens.

In MDR CNS isolates, all of them were resistant to the examined antibiotics apart from enrofloxacin. Similar to the report of Piessens *et al*. [37], 15.90% of CNS isolates from 82 mastitis cases were resistant to oxacillin, and only one isolate showed intermediate resistance to enrofloxacin. In this study, we found a high prevalence of MDR staphylococcus pattern with 46.67%, which is similar to the finding of Phophi *et al*. [36], who reported that more than half (51%) of CNS exhibited an MDR pattern, with primarily resistant to penicillin (88%) and ampicillin (85%). Numerous studies have indicated an increase in the prevalence of MDR staphylococcal bacteria, which result in chronic infection with varying degrees of prognosis depending on their antimicrobial resistance profile, presence of virulence factors, and biofilm-forming abilities. Coagulase-negative staphylococci biofilms provide protection against antibiotics or disinfectants. A significant proportion of penicillin-resistant CNS may be attributable to the widespread availability of these antimicrobials over the counter for the treatment of mastitis [38].

In the present study, we observed high clinical and bacteriological cure rates for parenteral amoxicillin against mastitis pathogens in clinical mastitis. This is consistent with the findings of Kalmus *et al*. [34], who showed that 77% were clinically cured and 55% were bacteriologically cured after benzylpenicillin treatment. The bacteriological cure rate was lower than the clinical cure rate. Clinical efficacy does not always indicate bacteriological efficacy. Although the clinical symptoms may have improved after treatment, pathogenic bacteria are identified. This could be due to recurrent and persistent MDR or biofilm-forming bacterial infection in the udder [39]. Parenteral amoxicillin treatment was the most effective against contagious and opportunistic bacterial infections, with a clinical and bacterial cure rate of 100%. Although being a mild acid, amoxicillin may penetrate the mammary gland poorly following parenteral administration. However, due to its low MIC value (0.12 mg/mL–32.00 mg/mL) against susceptible CNS [40], therapeutic concentrations in milk can be achieved and sustained through parenteral administration. This result was similar to the findings of Roberson *et al*. [15]. Similarly, previous report has confirmed that parental and IMM infusions with amoxicillin may be efficacious against most common Gram-positive mastitis pathogens, and most of them are labeled as efficacious against streptococci and staphylococci [37].

In subclinical cases, the effectiveness of treatment in the present study was mostly successful against *S. uberis*, followed by *Corynebacterium* spp., CNS, *S. agalactiae*, and *Streptococcus* spp. The explanation for why the susceptibility profile and treatment outcome of *Streptococci* and *Staphylococci* were different is subject to speculation but might be because the bacteria were maintained in a log phase of growth and could form a biofilm [34, 36]. Interestingly, although the results of antibiotic susceptibility tests in the laboratory showed that *Streptococcus* spp. were 100% susceptible to amoxicillin, the clinical and bacteriological cure rates were not very high in both clinical and subclinical mastitis cases. Because *in vitro* susceptibility does not always reflect therapeutic efficacy, it is difficult to predict clinical and bacteriological outcomes. Further studies must concentrate on the factors related to the outcomes of clinical and bacteriological mastitis, the increasing frequency of antibiotic resistance, and its effect on the bacteriological eradication of mastitis pathogens.

## Conclusion

The predominant microorganisms causing clinical and subclinical mastitis in dairy cows in the northern region of Thailand were *Streptococcus* and *Staphylococcus* spp. A dose of 15 mg/kg every other day for three doses of parenteral amoxicillin is generally successful in treating both clinical and subclinical mastitis caused by bacteria in the streptococcal and staphylococcal groups. Therefore, parenteral amoxicillin can be used to treat mastitis in dairy cows in both clinical and subclinical mastitis cases in veterinary practice in the upper region of Northern Thailand. Further studies should be conducted in each of the different regions.

## Authors’ Contributions

NP: Conceptualization of the study, carried out data acquisition, and drafted the manuscript. NP and AS: Performed fieldwork, implemented the study, and contributed to the drafting of the manuscript. MI and WS: Supervised the study, statistical analysis, and drafted the manuscript. WC: Data analysis and interpretation and drafted the manuscript. All authors have read, reviewed, and approved the final manuscript.
